# Identification of Mutation in Exon11 of *BRCA1* Gene in Bangladeshi Patients with Breast Cancer 

**DOI:** 10.31557/APJCP.2019.20.11.3515

**Published:** 2019

**Authors:** Latifa Nishat, Zinnat Ara Yesmin, Farida Arjuman, Sufi Hannan Zulfiqar Rahman, Laila Anjuman Banu

**Affiliations:** 1 *Department of Anatomy, Bangabandhu Sheikh Mujib Medical University, Shahbagh, *; 2 *Department of Histopathology, *; 3 *Department of Immunology and Molecular Biology, National Institute of Cancer Research and Hospital, Mohakhali, Dhaka, Bangladesh. *

**Keywords:** Exon11 mutation, BRCA1 gene, formalin fixed paraffin embedded (FFPE) breast cancer tissue

## Abstract

**Background:**

Worldwide, breast cancer is the leading cause of cancer death in female, in Bangladesh breast cancer is the second leading cancer in both sexes, and in women it occupied the top position. Highly penetrant mutations in *BRCA1* gene constitute high risk of breast cancer. The spectrum of *BRCA1 *gene mutations varies in different population. The objective of this study was to identify mutation in exon11 of *BRCA1* gene in Bangladeshi breast cancer patients.

**Methods:**

Genomic DNA was extracted from the histopathologically diagnosed formalin fixed paraffin embedded (FFPE) breast cancer tissues of 65 adult female patients. Two regions of exon11 of the *BRCA1* gene were amplified and the amplicons were sequenced using Sanger sequencing. The sequenced nucleotides were analyzed and blast using NCBI nucleoblast. Selected demographic, reproductive and medical histories were collected and analyzed using SPSS version 20.

**Results:**

The mean age of the patients was 46 years and the mean age at diagnosis was 44.64 years. The patients were married and had 2.65 ± 1.22 children except one was nulliparous, the mean age of menarche was 12.67 years. All patients had children, breastfed the babies for an average 1.5 years. Only 13.6% of the patients had hypertension and the rest had no comorbidity. The family history for cancer (breast and other cancer) was negative. Three novel mutations were found in a patient. Two among the three mutant sequences had effect on amino acid coding (DNA sequence change g.852G>C and g.709G>A and amino acid changes p.Gln284His and p.Glu237Lys respectively).

**Conclusion:**

We found three novel mutations in Bangladeshi breast cancer patients. This finding indicates the necessity to study the mutation profile of whole *BRCA1* gene in our population for cancer risk prediction**. **

## Introduction

Worldwide, breast cancer is the leading cause of cancer death in female, and about 2.1 million newly diagnosed breast cancer cases was estimated for the year 2018 (Bray et al., 2018). Hereditary and genetic factors including mutations in the *BRCA1* and *BRCA2 *genes and other breast cancer susceptible genes account for 5%–10% of breast cancer cases (Apostolou and Fostira, 2013). Breast cancer incidence rates are rising in the transitioned countries. Raised incidence rates in those countries are the effect of a increasing prevalence of the risk factors related to menstruation (early menarche, later age at menopause), reproduction (nulliparity, late age at first birth, and fewer children), exogenous hormone intake (oral contraceptive use and hormone replacement therapy), nutrition (alcohol consumption), and anthropometry (greater weight, weight gain during adulthood, and body fat distribution); while breastfeeding and physical activity are known protective factors (Bray et al., 2018).

Highly penetrant mutations in *BRCA1* gene constitute high risk of breast cancer (Bhatta et al., 2016). The spectrum of *BRCA1* gene mutations is different and has significant variation in their contribution to breast cancer in different population (Szabo and King, 1997). *BRCA1* is a large gene located in the long arm of chromosome 17 (17q12-21). The gene has twenty two exons coding a 220kD protein of 1,863 amino acids. The gene has a role in maintenance of genomic integrity and transcriptional regulation (Belogianni et al., 2004). *BRCA1* is characterized by a zinc-binding RING finger domain at the amino terminus and *BRCA1* carboxyl terminal (BRCT) domain at the carboxyl terminus (Zheng et al., 2000). Among the 22 exons, exon11 having 3426bp, is a large central exon of *BRCA1* gene and represents about 60% of the coding sequences (Khachibi et al., 2015). 

In Bangladesh, the reliable breast cancer statistics is lacking as there is no population-based cancer registries or a central cancer registry to provide comprehensive nationwide data. The only hospital-based cancer registry tracks new cancer cases systematically in Bangladesh at the National Institute of Cancer Research and Hospital (NICRH). According to an NICRH report, breast cancer was the second leading cancer (12.5%) in both sexes, and in women about 1363 new cases were diagnosed in 2014, which occupied the top position (27.4%) among female cancers (NICRH, 2015). The NICRH statistics stated the mean age of the breast cancer patients was 42.97 years, and majority of them (55%) are multipara (NICRH, 2015). Limited research has been carried out on breast cancer in Bangladesh. Therefore, the contribution of *BRCA1* gene mutations to breast cancer in Bangladeshi population remains relatively unexplored. Identification of *BRCA1 *gene mutation makes a great value in cancer prognosis, treatment and early risk prediction. This would help to reduce the risks efficiently before the occurrence of the disease and administer more individualized advanced therapies for better clinical responses (Dillenburg et al., 2012). Research using *BRCA1* gene mutation in breast cancer would help us in making assumptions on how this disease is transmitted from generation to generation. Thus, people having evidence of breast cancer in the family can be made aware of its occurrence in their future generation. Regarding Bangladeshi population this type of research is very limited to date. That’s why this study is carried out to identify* BRCA1* gene mutations in Bangladeshi female patients with breast cancer.

## Materials and Methods

The cross-sectional descriptive study was carried out in the Genetic Research Laboratory of the Department of Anatomy, Bangabandhu Sheikh Mujib Medical University (BSMMU), Dhaka from July 01, 2017 to June 30, 2018. Sixty-five histopathologically diagnosed formalin fixed paraffin embedded (FFPE) breast cancer tissue were collected from the National Institute of Cancer Research and Hospital (NICRH), Bangladesh. The demographic, reproductive, medical and family histories of the patients were collected using a structured questionnaire. 

*Isolation of DNA*

Genomic DNA was extracted from 5 (10-μm thick) sequential sections of histopathologically diagnosed formalin fixed paraffin embedded (FFPE) breast cancer tissue using commercial DNA extraction kit QIAamp DNA FFPE tissue kit (QIAGEN Gmbh, Germany). The tissue sections were taken in a 1.5 ml microcentrifuge tube and dissolved in xylene for removal of paraffin. The xylene-mixed tissue was centrifuged and supernatant was removed by pipetting. Residual xylene was removed by 96% ethanol. The residual ethanol was allowed to evaporate from the tissue by incubating the open tube at 37^o^C temperature for 10 minutes. The tissue was lysed under denaturing conditions with proteinase K. The lysate was incubated at 90^o^C for 1 hour to reverse the formalin cross linking. The DNA was isolated from the formalin-cross-linking free lysate using standard operation procedure (SOP) of the commercial kit. The extracted DNA samples were purified by RNase A (QIAGEN Gmbh, Germany). The RNA free DNA samples were tested qualitatively on 2% agarose gel and quantified by a nanodrop fluorometer. 

*Amplification*

Two sets (A and B) of forward and reverse primers (Table 1) were used to cover two hot spot regions of exon11 of *BRCA1* gene according to Singh (2015). 

**Figure 1:**
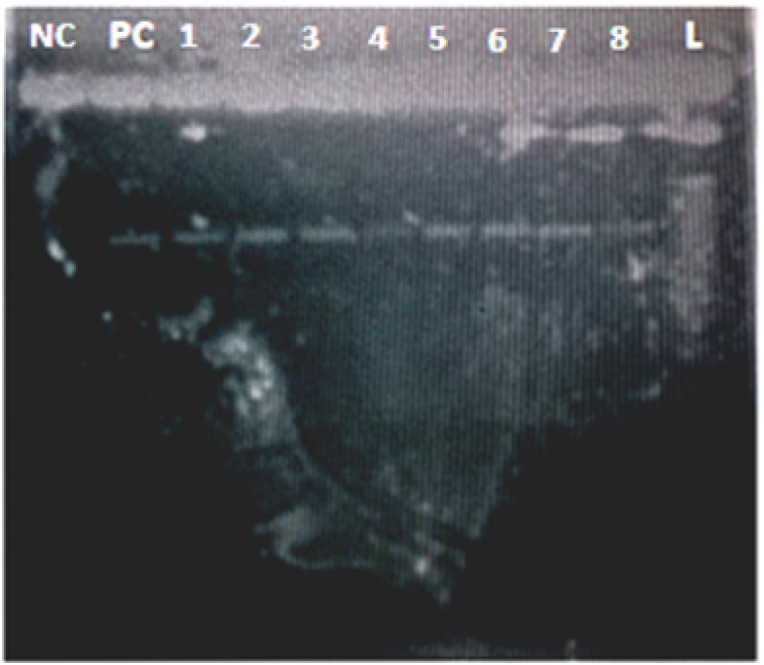
L, Ladder; PC, Positive control; NC, Negative control (water blank); samples 1-8 show band in 2% gel electrophoresis

The extracted DNA samples were amplified by short range PCR using the Hot Start Colourless Master Mix Kit Model: M7432 (Promega USA). Amplification was performed on a Biometra thermal cycler (Biometra GmbH, Germany). The amplicons were visually confirmed by 2% agarose gel electrophoresis (Figure 1). 130 (65X2) PCR products were sent for SANGER sequencing. 

*DNA sequencing*

In Cycle Sequencing the ABI-3500, BigDye terminator v 3.1 cycle sequencing ready reaction kit (Applied Biosystem, USA) was used which combine ampliTaq polymerase (Applied Biosystem, USA), the new BigDye terminator and all the required components for the sequencing reaction. In the ready reaction format the dye terminators, deoxy nucleotide triphosphate, ampliTaq DNA polymerase, magnesium chloride and buffer were premixed into a single tube of ready reaction mix and were ready to use. In this step only one strand of the desired DNA was amplified and the DNA fragment of different length that had been terminated with different ddNTPs were produced.

*Data analysis*

The sequenced data were analyzed by Chromas version 2.3 software and the nucleotide blast was done by NCBI nucleoblast. The sequences were analyzed using AB Applied Biosystems. The base sequences were matched with the reference sequences using Mega software. The altered sequences were checked for amino acids coding to see the effect on protein synthesis. 

*Ethical Implication*

All selected patients were informed that their DNA samples will be used for research purpose. They were also informed that they have the right to withdraw their participation from the study at any time. The study was approved by the Institutional Review Board of Bangabandhu Sheikh Mujib Medical University (BSMMU), and National Institute of Cancer Research and Hospital (NICRH), Bangladesh. A memorandum of understanding (MOU) was also signed by the concerned persons of the above-mentioned institutions.

**Table 1:** Description of Primers Used

Exon	Product size bp (base pair)	Primer set used	
		Forward primer	Reverse primer
Exon11 A	497	GACAATTCAGTTTTTGAGTACCTTG	TGTTATCCAAGGAACATCTTCAG
Exon11 B	436	CAGAAACTGCCATGCTCAGA	TATTTGTGAGGGGACGCTCT

**Table 2:** Mutation Report

DNA sequence change	Amino acid change (three-letter code)	DNA reference number	Site of mutation
g.852G>C	p.Gln284His	NM_007294.3(BRCA1)	Exon11
g.711A>G	p.Glu237Glu	NM_007294.3(BRCA1)	Exon11
g.709G>A	p.Glu237Lys	NM_007294.3(BRCA1)	Exon11

**Figure 2:**
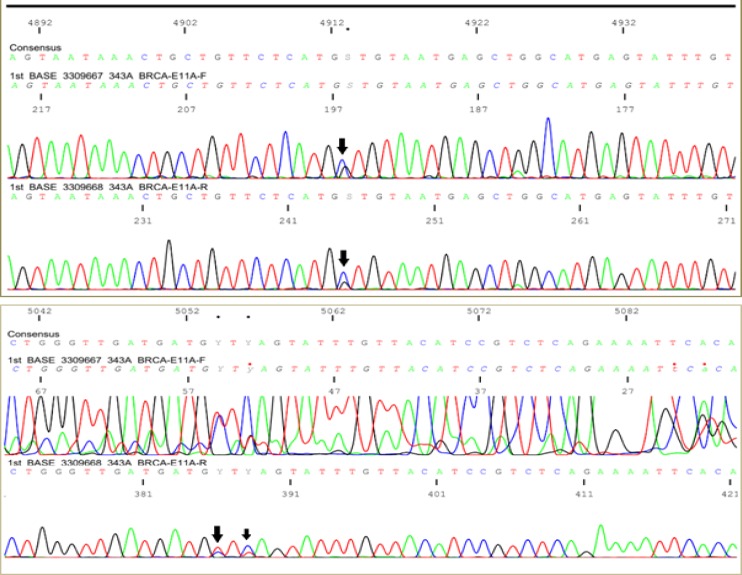
Sanger Sequence of Sample 343 Exon11 of Set A, the Arrow Showing Heterogenous Mutations

## Results

*Mutation results*

130 DNA samples (2 sets) sequences were analyzed using AB Applied Biosystems. Three novel mutations were found in a sample of DNA (GenBank accession number: BankIt2239534 343A_BRCA-E11A MN124391). The mutant sequences are presented in Figure 2 and the mutation report is in Table 2. 

*Demography of the patients*

Age and reproductive status of the participants were evaluated. The mean age of the patients was 46 years (range 34 to 57 years) and the mean age at diagnosis was 44.64 years (range 32 to 56 years). All the participants were married and had 2.65 ± 1.22 children except one was nulliparous, the mean age at menarche was 12.67 years (range 11 to 13 years). The mean age at marriage was 19.67 years (range 15 to 24 years), and the mean age at first delivery was 21.35 (range 17 to 26 years). All patients had children, breastfed their babies for an average 1.5 years. Only 13.6% of the patients had hypertension and the rest had no reported comorbidity. The family history for cancer (breast and other cancer) was negative in the study participants.

*Particulars of the BRCA1 mutation positive patient*

The *BRCA1* mutation positive patient was a married housewife having 4 children, 50 years of age, weight 55 kg and height was 160 cm (BMI 21.48kg/m^2^). She had menarche at 12 years of age, still menstruating with irregular cycles and 2 to 3 days duration, she had a history of abortion. She got married at 24 years of age and having her first child at 25 years, she breast fed all her children for about 2 years and she took oral contraceptives. She was diagnosed at 49 years of age and the cancer grade was locally invasive, estrogen and progesterone receptors were positive but HER2 negative, she had positive findings for breast cancer in both ultrasonography and mammography, fine needle aspiration (FNAC) biopsy found malignant cells. She was treated surgically followed by intravenous chemotherapy. She did not take tobacco in any form and had negative history of alcohol consumption. She was hypertensive and her family history for cancer was negative. 

## Discussion

We found three novel mutations in one hot spot of the *BRCA1* gene in a patient 50 years of age. Mutations in exon11 of *BRCA1* gene is heterogenous and may vary geographically, as the spectrum of *BRCA1* gene mutations is different and their contribution to breast cancer vary significantly in different population (Szabo and King, 1997). In the same population the mutation of *BRCA1* gene varies according to the age of onset of cancer. Singh (2015) observed a strong significant association of mutation of *BRCA1 *gene and the age at diagnosis in the North Indian women, *BRCA1* mutation was more frequently observed in tumor sample obtained from women who were ≤ 40 years of age (11.9%) than those >40 years old (1.2%). We found heterogenous mutations in *BRCA1* gene in a patient more than 40 years of age (age at diagnosis was 49 years). The mean age at diagnosis of our study population was 44.64 years and 3 sequences variants (4.6% mutation) in *BRCA1* gene were reported. The mean age at diagnosis of our patients is close to the mean age at testing of women of African descent (45.2 years) but lower than that of the Asian descent (47.1 years) and European women (50 years) (Hall et al., 2009). Bhatta (2016) reported the mean age of onset of breast cancer was 42.59 years and found 8% of *BRCA1 *mutation in his study population in Nepal. Another study in Nepal reported older age (mean age 46.50 years) of onset (Singh and Sayami, 2009) than that of Bhatta (2016). The mean age of onset was 47.50 years in the North Indian women (Singh et al., 2015) is higher than the age at diagnosis of our population. High rate (16.3%) of mutation in *BRCA1* gene was reported in an Indian study (Karami and Mehdipour, 2013) whereas another study on the Eastern Indian reported no mutation (Chakraborty et al., 2014). Our study population is more similar to that of Chakraborty et al., (2014), as they share same ethnicity (Bengali) and many socio-cultural factors. 

Apostolou and Fostira (2013) claimed hereditary and genetic factors including mutations in the *BRCA1* and *BRCA2* genes and other breast cancer susceptible genes account for 5%–10% of breast cancer cases. Studies in transitioned countries found that the increased incidence rates of breast cancer are the result of a higher prevalence of menstruation related risk factors (early age at menarche, later age at menopause), risk factors related to reproduction (nulliparity, late age at first birth, and fewer children), exogenous hormone intake (oral contraceptive use and hormone replacement therapy), nutrition (alcohol intake), and anthropometry (greater weight, weight gain during adulthood, and body fat distribution); and breastfeeding and physical activity are marked as protective factors (Bray et al., 2018). The economy of Bangladesh is fast growing as we achieved lower-middle income country from the low income status (World Bank, 2019); our females actively participate in this process. In this contrast, we have the same risk factors for increasing incidence of breast cancer as found in the transitioned country. But the reproductive history of our patients was different from that of the transitioned countries. The mean age at menarche was 12.67 years in our patients with breast cancer. They had fewer numbers of children (2.65 ± 1.22) but early age at marriage (mean 19.67 years), and the mean age at first delivery was 21.35 years in our study population. All patients had children, breastfed their babies for an average 1.5 years. History of smoking and alcohol consumption was negative in our patients. The only similar risk factors in transition country like late menopause and use of oral contraceptives are positive in our *BRCA1* gene mutation positive patient. The result of increasing new risk factors related to reproductive history will take time to increase incidence of breast cancer.

In this study, we found three novel mutations in one sample of FFPE tissue. Gornjec (2019) detected mutations of *BRCA1* and *BRCA2* genes in FFPE tissue, cytological samples and blood cells and found 100% concordance in detection of germline mutations in these samples. That study also found two somatic mutations in FFPE tissue and cytological samples and there was 100% concordance between these samples. As we extracted DNA only from FFPE tissue, we cannot say whether these mutations are germline or somatic.

The variation in our study may be due to small reference population we included. In addition it may also be possible that the mutation may be occurring in other areas of exon11 or other exons of *BRCA1* gene, or even in other genes like BRCA2, p53 etc. which thus initiated an importance to screen those mutation as well. 

In Conclusion, mutation of *BRCA1* gene varies among population and age of onset of cancer. We found three novel mutations in Bangladeshi breast cancer patients. This finding indicates the necessity to study the mutation profile of whole* BRCA1* gene in our population for cancer risk prediction. The analysis of risk factors related to reproduction and lifestyle and recent trend of economy, forecasts increase incidence of breast cancer in near future in Bangladesh. 
